# The Association of Hemoglobin A1c and Fasting Glucose Levels with hs-CRP in Adults Not Diagnosed with Diabetes from the KNHANES, 2017

**DOI:** 10.1155/2021/5585938

**Published:** 2021-04-01

**Authors:** Jeong Woo Seo, Sat Byul Park

**Affiliations:** Department of Family Practice and Community Health, Ajou University School of Medicine, Suwon, Republic of Korea

## Abstract

**Purpose:**

High sensitivity C-reactive protein (hs-CRP) has been used as a biomarker to assess the risk of cardiovascular accidents (CVA) and to measure general inflammation in the body. This study investigated the relationship and extent of correlation between serum glucose level markers and hs-CRP as a means to assess CVA risk through hemoglobin A1c (HbA1c) and fasting glucose levels.

**Methods:**

This cross-sectional, population-based study used data from the 2017 Korea National Health and Nutrition Examination Survey (KNHANES). From the total sample of 8,127 people, 4,590 subjects were excluded due to age (<19 years) (*n* = 1,505), diabetes mellitus (DM) diagnosis or medication (*n* = 596), inactivity (*n* = 424), pregnancy (*n* = 17), hypoglycemia (<70 mg/dL) (*n* = 8), smoking history (*n* = 1,077), and missing data (*n* = 963). In total, 3,537 adults not diagnosed with diabetes were selected. Their hs-CRP levels were compared with the glucose level markers using a complex sample general linear regression analysis.

**Results:**

We adjusted for sedentary hours, smoking, binge drinking frequency, age, sex, mean SBP, triglycerides, and waist circumference. Increases in HbA1c correlated with hs-CRP levels (*B* coefficient (95%CI) = 0.185, *p* = 0.001, and *R*^2^ = 0.087). Changes in the fasting glucose levels were also associated with the hs-CRP levels (*B* coefficient (95%CI) = 0.005, *p* = 0.006, and *R*^2^ = 0.086).

**Conclusion:**

This study showed a linear association between HbA1c and fasting glucose levels and hs-CRP. It also showed that changes in the hs-CRP level were better correlated with those in the HbA1c levels than in the fasting glucose levels.

## 1. Introduction

C-reactive protein (CRP) is an acute-phase reactant, and its circulating concentration rises rapidly as a cytokine-mediated response to tissue injury, infection and inflammation [1]. While CRP is measured within the range of 10 to 1,000 mg/L, high sensitivity-CRP (hs-CRP) is measured in the range of 0.5 to 10 mg/L [2]. CRP has inflammatory activity; pentameric CRP changes into monomeric CRP and activates the complement system and inflammatory cells, such as monocytes, in the endothelium. The process results in the formation of atherosclerotic plaques. Chronic CRP elevations may have biologic effects on endothelial function, coagulation, fibrinolysis, oxidation of low-density lipoproteins (LDL), and atherosclerotic plaque stability [3]. Some studies have suggested that higher CRP levels are associated with increased risk of a cardiovascular event [4]. The CRP level is also associated with the cardiovascular event-free survival rate [5]. The American Heart Association (AHA) has established that cardiovascular risk is dependent on hs-CRP levels [6].

HbA1c levels also show concentration-dependent elevation with the increase in cardiovascular risk. Studies have suggested that subgroups show the same trend [7]. Fasting glucose levels above and below certain limits are also associated with increased risk of a cardiovascular event [8]. Some studies have examined the relationship between HbA1c, fasting glucose, and hs-CRP in diabetic patients [9], but this association has rarely been studied in undiagnosed diabetes cases in Korea.

The current study was conducted to determine whether HbA1c and fasting glucose levels correlate with hs-CRP in Koreans with undiagnosed diabetes, as well as to determine which of these parameters better reflects cardiovascular risk.

## 2. Methods

### 2.1. Trial Population

**Figure 1:**
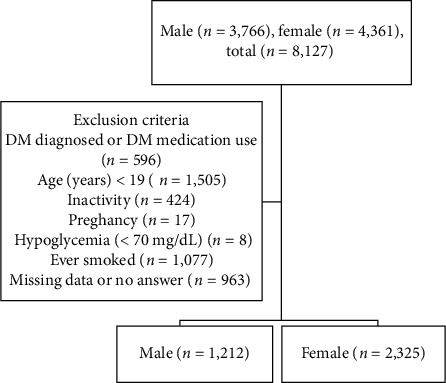
Flow chart on inclusion process of study population.

This study used data from the 2017 Korea National Health and Nutrition Examination Survey (KNHANES). We collected data from Korean adults aged 19–80. We excluded subjects diagnosed with or receiving medication for diabetes, who had a disability, were pregnant, hypoglycemic (under 70 mg/dL), had a history of smoking, or had not provided complete data. Finally, targeted data were obtained from 1,212 males and 2,325 females (Figure 1).

### 2.2. Analysis Variables

The AHA suggests that atherosclerosis is a multifactorial disease involving factors related to high blood pressure, smoking, dyslipidemia, gender, age, inactive lifestyle [10], and binge drinking [11]. Hence, these variables were included for adjustment. In the current study, hs-CRP, HbA1c, fasting glucose, age, waist circumference, mean systolic blood pressure, daily sedentary hours, triglycerides, and white blood cell (WBC) count were measured as continuous variables, while gender, binge drinking frequency, and smoking were considered as nominal variables. Mean systolic blood pressure was calculated as an average of the second and third blood pressure readings taken from each subject.

### 2.3. Statistical Analysis

The continuous variables in the baseline characteristics were analyzed using a *t*-test and the nominal variables with a chi-square analysis. The associations between HbA1c and fasting glucose levels and hs-CRP were analyzed using a complex sample general linear regression analysis. IBM SPSS Statistics ver. 21.0 (IBM Co., Armonk, NY, USA) was used, and the results were accepted as statistically significant if the *p* values were less than 0.05.

## 3. Results

### 3.1. Basal Characteristics

Participants' basal characteristics are shown in Table 1. hs-CRP and fasting glucose levels were higher in males than in females, and the differences were statistically significant (*p* < 0.05). The HbA1c level was not statistically different between the sexes. Mean SBP, triglyceride, and smoking rates were also statistically higher among males than among females.

### 3.2. Association between hs-CRP and HbA1c and Fasting Glucose Levels

The association between hs-CRP and HbA1c and fasting glucose was examined in the unadjusted condition in Model 1. Other variables that could affect atherosclerosis (age, gender, mean SBP, triglycerides, sedentary hours a day, binge drinking frequency, waist circumference, and smoking status) were included in Model 2. In Model 3, the WBC count was added to Model 2.

hs-CRP levels increased significantly with HbA1c increases in both sexes in Model 1 (*B* coefficient (95%CI) = 0.440, *p* < 0.001, and *R*^2^ = 0.020), and this trend remained consistent in Model 2 (*B* coefficient (95%CI) = 0.229, *p* < 0.001, and *R*^2^ = 0.062) and Model 3 (*B* coefficient (95%CI) = 0.185, *p* = 0.001, and *R*^2^ = 0.087).

Fasting glucose showed a lower regression coefficient than HbA1c in all models. hs-CRP levels increased significantly as fasting glucose levels increased in Model 1 (*B* coefficient (95%CI) = 0.013, *p* < 0.001, and *R*^2^ = 0.016). In Models 2 and 3, the association between fasting glucose and hs-CRP was weaker (*B* coefficient (95%CI) = 0.005, *p* = 0.004, and *R*^2^ = 0.059 in Model 2; *B* coefficient (95%CI) = 0.005, *p* = 0.006, and *R*^2^ = 0.086 in Model 3) (Table 2). Both models showed a similar trend in which the regression coefficients were lower in the adjusted models, but the decision coefficient was higher and its explanatory power increased.

The association between HbA1c and hs-CRP showed a persistent trend through all adjustment models in both sexes. There was also a correlation between fasting glucose and hs-CRP levels in females. However, when analyzing the association among males, there was no statistical significance in Models 2 and 3 (Tables 3 and 4).

## 4. Discussion

The HbA1c-hs-CRP models have higher regression coefficients than the fasting glucose-hs-CRP models. These results suggest that the HbA1c levels better reflect the hs-CRP levels than the fasting glucose levels by the absolute *B* coefficient. There was no specifically different trend between the male and female models. HbA1c could be used as a prognostic marker of atherosclerosis in cases of undiagnosed diabetes. There are few studies about the association between these two parameters in patients with undiagnosed diabetes in Korea. Therefore, based on the results of this study, subclinical cardiovascular risks can be explained in some clinical settings.

In 2016, 14.4% (approximately 5.02 million) of Korean adults had diabetes. The prevalence rate of impaired fasting glucose was 25.3% (8.71 million) [12]. Among Korean adults, the overall prevalence of clinical atherosclerotic cardiovascular disease (ASCVD) per 1,000 individuals was 98.25 in 2014 and 101.11 in 2015 [13]. It has, therefore, become important to control diabetes-related and cardiovascular risks in Koreans, whether or not the patients have specific chronic illnesses.

Previous studies have explored the association between serum glucose and inflammation. Some studies have suggested that reactive oxidative species from glycation end products are a proinflammatory effect of increased glucose levels [14]. Another plausible mechanism is that hyperglycemia affects NF-*κ*B, a key mediator that regulates multiple proinflammatory and proatherosclerotic target genes in endothelial cells, vascular smooth muscle cells, and macrophages [15, 16]. A high association was found between HbA1c and hs-CRP levels in this study, despite the fact that CRP is an acute reactant and HbA1c is a measure of the glycated hemoglobin over a period of 3–4 months, which is the average life span of red blood cells [17]. This mechanism requires further study.

This study had some limitations. First, as less data were available about LDL cholesterol, triglycerides were chosen as a measure of the dyslipidemia effect. Hence, more precise analyses with LDL cholesterol adjustment may be warranted in future studies. Second, former smokers were excluded from the sample to avoid any effects from past smoking. Hence, the number of years of smoking could also be an important variable affecting the adjusted results. Third, because this was a cross-sectional study, prospective trials are needed to further determine the demonstrated associations.

In conclusion, the results indicated a linear association between HbA1c and fasting glucose levels and hs-CRP levels. It was also shown that the change in the hs-CRP level is better correlated with the HbA1c level than with the fasting glucose level.

## Figures and Tables

**Table 1:** Baseline characteristics for participants.

	Male (*n* = 1,212)	Female (*n* = 2,325)	*p* value
Age (years)	44.19 ± 0.45	49.41 ± 0.33	<0.001
Waist circumference (cm)	85.35 ± 0.27	77.45 ± 0.20	<0.001
Mean SBP (mmHg)	119.45 ± 0.43	115.94 ± .037	<0.001
Sedentary hours a day	8.20 ± 0.11	7.97 ± 0.07	0.052
hs-CRP (mg/L)	1.28 ± 0.06	1.01 ± 0.03	<0.001
Fasting glucose (mg/dL)	98.35 ± 0.55	94.80 ± 0.28	<0.001
HbA1c (%)	5.52 ± 0.02	5.52 ± 0.01	0.887
Triglycerides (mg/dL)	163.64 ± 4.02	108.87 ± 1.50	<0.001

Binge drinking frequency			<0.001
None	368 (10.4%)	1,670 (47.2%)	
≤1/month	203 (5.7%)	314 (8.9%)	
1/month	226 (6.4%)	188 (5.3%)	
1/week	277 (7.8%)	132(3.7%)	
Always	138 (3.9%)	21 (0.6%)	

SBP: systolic blood pressure. Values are presented as mean ± standard error. Mean and standard error are estimated values reflecting the complex sample weight. *p* values represent differences between men and women by *t*-test for continuous variables and chi-square for nominal variables.

**Table 2:** Complex samples of general linear model for HbA1c, fasting glucose, and hs-CRP in the study subjects.

	HbA1c				Fasting glucose			
	*B* coefficient (95% CI)	SE	*p* value	_*R*_^2^	*B* coefficient (95% CI)	SE	*p* value	_*R*_^2^
Model 1	0.440	0.052	<0.001	0.020	0.013	0.002	<0.001	0.016
Model 2	0.229	0.056	<0.001	0.062	0.005	0.002	0.004	0.059
Model 3	0.185	0.087	0.001	0.087	0.005	0.002	0.006	0.086

*β* coefficients are estimated values in mg/dL reflecting the complex sample weight. Model 1 on both categories is the unadjusted model. Model 2 is adjusted by age, gender, mean SBP, triglycerides, sedentary hours a day, binge drinking frequency, waist circumference, and smoking state. Model 3 is added by adjusting WBC in Model 2. SE: standard error.

**Table 3:** Complex samples of general linear model for HbA1c and hs-CRP in males and females.

	Male				Female			
	*B* coefficient (95% CI)	SE	*p* value	_*R*_^2^	*B* coefficient (95% CI)	SE	*p* value	_*R*_^2^
Model 1	0.428	0.093	<0.001	0.017	0.449	0.061	<0.001	0.023
Model 2	0.255	0.101	0.012	0.042	0.207	0.067	0.002	0.073
Model 3	0.205	0.100	0.041	0.068	0.177	0.066	0.011	0.097

*β* coefficients are estimated values in mg/dL reflecting the complex sample weight. Model 1 on both categories is the unadjusted model. Model 2 is adjusted by age, gender, mean SBP, triglycerides, sedentary hours a day, binge drinking frequency, waist circumference, and smoking state. Model 3 is added by adjusting WBC in Model 2. SE: standard error.

**Table 4:** Complex samples of general linear model for fasting glucose and hs-CRP in males and females.

	Male				Female			
	*B* coefficient (95% CI)	SE	*p* value	_*R*_^2^	*B* coefficient (95% CI)	SE	*p* value	_*R*_^2^
Model 1	0.010	0.003	<0.001	0.010	0.015	0.002	<0.001	0.019
Model 2	0.005	0.003	0.101	0.039	0.005	0.002	0.029	0.071
Model 3	0.005	0.003	0.112	0.066	0.005	0.002	0.035	0.096

*β* coefficients are estimated values in mg/dL reflecting the complex sample weight. Model 1 on both categories is the unadjusted model. Model 2 is adjusted by age, gender, mean SBP, triglycerides, sedentary hours a day, binge drinking frequency, waist circumference, and smoking state. Model 3 is added by adjusting WBC in Model 2. SE: standard error.

## Data Availability

The authors confirm that the data supporting the findings of this study are available within the article. Raw data were generated at https://knhanes.cdc.go.kr. Derived data supporting the findings of this study are available from the corresponding author on request. There are no restrictions on data access.
